# Toothaches in the Daily Lives of Brazilian Adults

**DOI:** 10.3390/ijerph9082587

**Published:** 2012-07-25

**Authors:** Aline Mendes Silva de Pinho, Ana Cristina Campos, Efigênia Ferreira, Andréa Maria Duarte Vargas

**Affiliations:** Department of Social and Preventive Dentistry, School of Dentistry, Federal University of Minas Gerais, Avenida Presidente Antônio Carlos, 6627 Pampulha, Belo Horizonte, Minas Gerais CEP 31270-901, Brazil; Email: hannakaxyss@hotmail.com (A.C.C.); efigenia@uai.com.br (E.F.); vargasnt@task.com.br (A.M.D.V.)

**Keywords:** oral health epidemiology, adult, toothache, prevalence, impact, dental public health

## Abstract

The aim of the present study was to estimate the prevalence of toothaches and to evaluate its effects on the daily lives of adults living in an industrialised region of southeastern Brazil. A questionnaire was administered to a sample of 744 individuals. The variables related to toothache were grouped into three components: access to dental service, pain severity, and social/functional impacts. The present study found that 68.0% of the subjects had limited access to oral health care, 39.7% presented high toothache severity, and 47.3% reported that toothache greatly affected their daily lives. Nervousness (87.2%) and chewing difficulty (72.6%) were the most commonly reported toothache-related effects. Through correspondence analysis, four groups with separate profiles for toothache and associated factors were identified. Two groups reported greater effects of toothaches in their daily lives. One group consisted of individuals who had less access to dental services (women and individuals who were multiracial, married, had a middle school education, or a low family income). The other group consisted of individuals who reported a high toothache severity and high degree of social/functional impacts (individuals who were 40 to 44 years old, married or widowed, black or multiracial, and had a middle school education). The other two groups were those whose daily lives were less affected by toothaches. One group consisted of individuals who had greater access to dental services (men and individuals who were divorced, had a college degree, or had incomes greater than R$ 300.01). The final group consisted of individuals who had low toothache severity and a low degree of associated social/functional impacts (individuals who were 35 to 39 years old, white, single, or had a high school education).

## 1. Introduction

According to the International Association for the Study of Pain (IASP), pain is an unpleasant sensory and emotional experience associated with a real or potential tissue injury [1]. Pain occurs throughout the lifespan of humans and is one of the great challenges of science because of its numerous implications. When an individual is experiencing pain, he or she may exhibit altered behaviour and experience changes in daily routine activities [2]. Oral diseases can cause pain, suffering, embarrassment, and social hardship resulting in losses at the individual and collective levels [3].

Oral health problems are increasingly recognised as significant causes of negative effects on daily performance and quality of life at both the individual and community level [4]. Toothache is one of the most frequent sources of pain in humans; because of failures to manage the social determinants and materials used to promote oral health, millions of people suffer from toothaches and an associated low quality of life [5]. In addition to being a source of physical and emotional stress, toothache significantly affects quality of life and represents a substantial economic burden to society because of high treatment costs and loss of productivity [6].

In Brazil, toothache is one of the main reasons people seek dental care. The most recent national data shows a 27% prevalence of toothache among Brazilian adults 35 to 44 years [7]. Toothache and its psychosocial impact can be valuable indicators of oral health, complementing the clinical evaluation and extending the understanding of health through a subjective, behaviour-based perspective [8]. The aim of the present study was to estimate the prevalence of toothache among adults living in an industrialised region of southeastern Brazil and correlate it with information regarding access to care, pain severity, and social and functional limitations in daily life.

## 2. Methods

A cross-sectional epidemiological study was conducted in an industrialised region of southeastern Brazil from September to December 2010. The study population consisted of men and women aged 35 to 44 years living in the urban area of a large city. The Ethics and Research Committee of the Federal University of Minas Gerais (Comitê de Ética e Pesquisa da Universidade Federal de Minas Gerais) approved the present study under Protocol No. 096/2009.

As cluster sampling was employed, the sample size calculation considered the prevalence of toothaches among Brazilian adults (2004; 34.8%). The total adult population of the city of Betim (378,089 inhabitants) and the number of adults aged 35 to 44 years (56,661), as reported by the Brazilian Institute of Geography and Statistics (IBGE, 2004), were included in the calculation. With an expected error was 5%, confidence level of 95%, and a 20% compensation for loss and correction of the sampling design (deff), the samples included a total of 838 individuals.

The sample selection process was conducted in three phases: census tracts, residential blocks and households. The census tract is the smallest sampling unit used in the Brazilian census. Each census tract has approximately the same number of households (300) [9]. Ten census tracts, 58 residential blocks, and an average of 25 households per block were randomly selected. Approximately 1,450 households were randomly selected to achieve the required sample size. All individuals living in households that met the study’s eligibility criteria were selected.

Data were collected via a questionnaire and clinical-epidemiological examination performed by five previously trained and calibrated surgeon-dentists. The test-retest of the questionnaire in a group of 50 people was performed with a 15-day interval. For ordinal variables, agreement measures were above 0.70 (weighted kappa with 95% CI = 0.60 to 0.82). The intraclass correlation coefficient (ICC) was calculated for toothache duration in days, (ICC = 0.60, 95% CI = 0.56 to 0.72). A pilot study involving 98 people was conducted to test the methodology and the instrument to be applied.

The dependent variable investigated was toothache occurring within six months prior to the interview, as determined by the question "Have you experienced a toothache in the last six months?" The independent variables and categories used were as follows: education (illiterate, completed middle school, completed high school, and college); income (investigated as a continuous variable, income in Brazilian reais (R$), and then categorised according to the median, ≤R$ 300.00 and >R$ 300.01); self-reported skin colour (white, black, multiracial, and other), age (35 to 39 years and 40 to 44 years); and marital status (married/cohabitating, divorced/legally divorced, and widowed/single).

A survey adapted from the Dental Impact on Daily Living (DIDL) [10] was generated. The questions were grouped into three components defined by epidemiological basis, access to dental services, pain severity, and social/functional impacts.

The variables related to access to dental services were categorised as follows: regular dental appointments (yes/no); type of service sought (public/private/other); visited a dentist in the last six months (yes/no); problems with access (yes/no); frequency of dental visits (up to one visit/two or more visits); reason for dental visit (pain/prevention/treatment); type of services sought for emergency care (public/other ); and difficulty accessing emergency care (yes/no).

To assess pain severity, the following variables were used: pain intensity (weak, somewhat strong, strong, and intolerable) and duration of pain (<1 day, 2 to 3 days, 4 to 7 days, and >8 days). To evaluate social/functional limitations, the following variables were used: pain when eating sweets, pain when eating cold foods, pain when eating hot foods, spontaneous pain, pain when chewing, pain that prevents eating, pain that prevents sleeping or resting, pain that prevents tooth brushing, pain that prevents smiling, pain that prevents having fun, pain that makes the individual nervous, and loss of working hours because of pain. These variables were evaluated in dichotomously (yes/no).

To calculate the score for each component, the answers were weighted with higher values for responses that indicated a greater impact. These values were summed and divided by the number of questions in the respective component. The values obtained for each component were converted to a scale from 0 to 100 using a simple rule of three. Two groups in the access to dental service component (reduced access and increased access), the pain severity component (low severity and high severity) and the social/functional impacts component (low impact and high impact) were established and categorised according to the median value.

Cronbach’s alpha coefficient was used to confirm the internal consistency of these issues in each of the defined variables. The same reliability was found on the subscales and total scale. 

The database was constructed and analysed using the Statistical Package for the Social Sciences (SPSS) software, Version 19. As cluster sampling was employed, the statistical analyses were performed considering the sample weights for each selection level of the sample (census tracts, residential blocks and households). A descriptive analysis of all study variables was performed.

Subsequently, we sought to explore the relationships between the established components (social/functional limitation, pain severity, and access to care) and the participants’ socioeconomic and demographic characteristics via a multiple correspondence analysis. Multiple correspondence analysis is an exploratory technique used to analyse categorical data with many variables and to graphically view groups with similar profiles.

## 3. Results

Of the 838 individuals selected to participate in the study, 744 (88.78%) were examined and interviewed. The nonparticipants (11.22%) refused to participate in the study or were excluded due to edentulism. All questions obtained values above 0.6 (range: 0.663 to 0.723), which showed a good reliability of the questions and the groupings. The Cronbach’s alpha total scale was 0.70, which is also considered satisfactory. The results of Cronbach’s alpha coefficient scale are shown in Table 1.

**Table 1:** Scale analysis for each component of toothache and its respective questionnaire items according to Cronbach’s alpha analysis.

Variables	Cronbach’s alpha (per item)	Cronbach’s alpha (per domain)	Cronbach’s alpha (total)
Access to dental service			
Emergency service sought	0.723	α = 0.62	
Difficulty accessing emergency care	0.707		α = 0.70
Pain severity			
Pain intensity	0.686	α = 0.60	
Pain duration	0.704		
Social/functional impacts			
Sweet foods	0.698	α = 0.81	
Cold foods	0.688		
Hot foods	0.676		
Spontaneous pain	0.690		
Pain when chewing	0.694		
Stopping eating	0.670		
Stopping sleeping	0.663		
Stopping brushing teeth	0.679		
Stopping laughing	0.683		
Nervousness	0.685		
Stopping having fun	0.671		
Stopping working	0.674		

The toothache prevalence was 24.3%, Confidence Interval (CI) was 21.1 to 27.9%. Demographic data for the sample are shown in Table 2.

**Table 2:** Demographic data of the sample of Brazilian adults, 2010.

Variables	N	%	95% CI
**Age group (N = 744)**			
35 to 39 years old	387	52.0	49.9–57.8
40 to 44 years old	357	48.0	42.2–50.1
**Gender (N = 744)**			
Male	229	30.8	28.4–35.8
Female	515	69.2	64.2–71.6
**Self-reported skin colour (N = 744)**			
White	187	25.1	20.2–26.9
Multiracial	370	49.7	46.9–54.8
Black	94	12.6	10.0–15.3
Other	25	3.4	2.4–5.5
**Marital status (N = 737)**			
Married/cohabitating	534	72.5	68.5–75.7
Separated/legally separated/divorced	60	8.1	6.1–10.5
Widowed	13	1.8	1.0–3.3
Single	130	17.6	15.0–21.1
Did not answer	6		
**Family income *per capita* (in R$) (N = 744)**			
≤300.00	385	54.4	52.6–60.6
>300.01	323	45.6	39.4–47.4
**Education (N = 744)**			
Illiterate	20	2.7	1.8–4.7
Middle school (up to 4 years of school)	414	56.0	53.8–61.6
High school (5 to 11 years of school)	251	34.0	28.1–35.4
College (more than 11 years of school)	54	7.3	5.8–10.1
**Regular dental visits (N = 744)**			
Yes	262	36.3	30.7–38.2
No	459	63.7	61.8–69.3
**Type of health care (N = 744)**			
Public service	216	29.6	27.7–35.2
Private office	392	53.7	48.7–56.7
Other	122	16.7	13.3–19.9
**Dental visit in the past year (N = 744)**			
Yes	414	56.8	52.2–60.1
No	316	43.2	39.9–47.8
**Reason for dental visit (N = 744)**			
Pain	183	27.2	23.9–31.2
Prevention	169	25.1	18.6–25.0
Treatment	322	47.8	38.2–46.0

Regarding pain duration, the average number of days with symptoms was 1.84 (±2.68). Data regarding access to dental service, pain severity, and the social/functional impacts of toothaches are shown in Table 3.

**Table 3:** Access to dental services, pain severity, and social/functional impacts in the adult population with toothaches, 2010.

Variables	N	%	95% CI
Access to dental service			
**Services sought for emergency care (N = 172)**			
Private	78	49.4	39.1–55.9
Public	64	40.5	34.8–51.5
Other	16	10.1	5.8–15.7
Did not answer	14		
**Difficulty accessing emergency care (N = 172)**			
No	74	57.4	46.9–65.0
Yes	55	42.6	35.0–53.1
Did not answer	43		
**Pain severity**			
**Toothache intensity (N = 172)**			
Weak	42	25.1	17.2–30.4
Slightly strong	32	19.2	15.7–29.5
Strong	58	34.7	26.9–42.6
Intolerable	35	21.0	14.8–28.4
Did not answer	5		
**Toothache duration (in days) (N = 172)**			
≤1	36	25.7	19.4–35.4
2 to 3	48	34.3	27.4–44.4
4 to 7	34	24.3	15.4–29.6
8+	22	15.7	10.7–23.9
Did not answer	32		
Social/functional impacts			
**Pain when eating sweet foods (N = 172)**			
No	70	41.7	32.3–48.2
Yes	98	58.3	51.8–67.7
Did not answer	4		
**Pain when eating cold foods (N = 172) **			
No	55	32.7	25.8–41.2
Yes	113	67.3	58.8–74.2
Did not answer	4		
**Pain when eating hot foods (N = 172)**			
No	72	42.9	31.9–47.6
Yes	96	57.1	52.4–68.1
Did not answer	4		
**Spontaneous pain when eating (N = 172)**			
No	55	32.9	22.9–37.7
Yes	112	67.1	62.3–77.1
Did not answer	5		
**Pain on chewing (N = 172)**			
No	46	27.4	22.2–37.2
Yes	122	72.6	62.8–77.8
Did not answer	4		
**Pain preventing eating (N = 172)**			
No	56	33.7	23.6–38.2
Yes	110	66.3	61.8–76.4
Did not answer	6		
**Pain preventing sleeping or resting (N = 172)**			
No	54	37.0	26.0–42.1
Yes	92	63.0	57.9–74.0
Did not answer	26		
**Pain preventing tooth brushing (N = 172)**			
No	70	47.3	39.0–56.3
Yes	78	52.7	43.7–61.0
Did not answer	24		
**Pain preventing smiling and showing teeth (N = 172)**			
No	71	48.0	38.8–56.1
Yes	77	52.0	43.9–61.2
Did not answer	24		
**Pain that made the subject nervous (N = 172)**			
No	19	12.8	7.3–18.4
Yes	129	87.2	81.6–92.7
Did not answer	24		
**Pain preventing having fun with other people (N = 172)**			
No	55	37.2	26.5–42.7
Yes	93	62.8	57.3–73.5
Did not answer	24		
**Pain preventing working (N = 172)**			
No	88	61.1	49.3–66.7
Yes	56	38.9	33.3–50.7
Did not answer	28		

Regarding the components of toothache, 68.0% of the subjects had low access to emergency oral health care services through public, private, or health insurance providers. Regarding pain severity, 39.7% of the studied subjects had experienced intense pain and a higher number of days with toothaches compared with the other participants. Forty-seven percent (47.3%) of respondents reported that toothache had a high impact on their social/functional abilities. Other results are shown in Table 4.

**Table 4:** Scale of the components of toothache in Brazilian adults, 2010.

Components	N	%
**Access to dental services **		
≤60.0 (low access)	83	68.0
>61.0 (high access)	39	32.0
**Pain Severity **		
≤62.5 (low severity)	82	60.3
>62.6 (high severity)	54	39.7
**Social/functional impacts**		
≤80.77 (low impact)	69	52.7
>80.78 (high impact)	62	47.3

**Figure 1:**
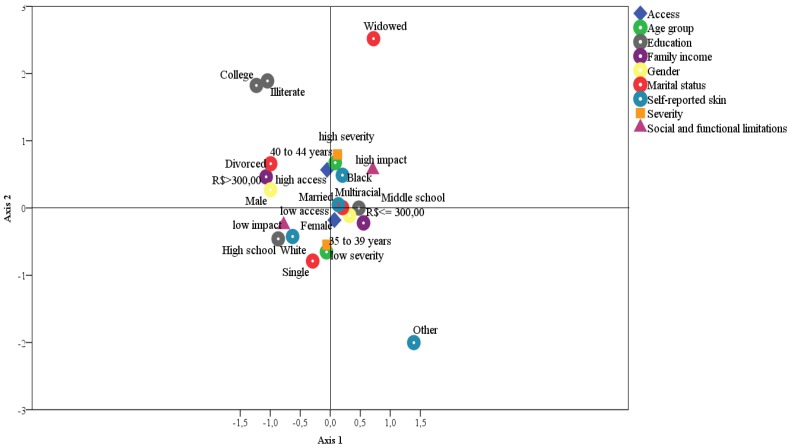
Score categories related to dental pain and the socioeconomic and demographic characteristics in Brazilian adults, derived from the correspondence analysis, 2010.

The results of correspondence analysis are illustrated in Figure 1. Four groups with different profiles for toothache and associated factors were identified. Two groups showed greater effects of toothache in daily life. The first group consisted of individuals who had less access to oral health care services (women and individuals who were multiracial or non-white, married, had a middle school education, and had the lowest incomes); the other group consisted of individuals who reported a high toothache severity and a high impact on social/functional limitations (individuals who were 40 to 44 years old, married or widowed, black or multiracial, and had completed middle school). The other two groups were those for whom toothache had less impact on daily life. One group consisted of individuals who had better access to oral health care services (men and individuals who were divorced, were illiterate or had a college degree, and earned more than R$ 300.01); the other group consisted of individuals who had low severity and low social and functional limitation (individuals aged 35 to 39 years who were white, single, and had a high school diploma).

## 4. Discussion

This is a cross-sectional study conducted in an industrialised region of southeastern Brazil that used a representative sample based on standardised criteria that was previously tested in large population studies [7].

In the present study, the prevalence of toothaches in the last six months among adults 35 to 44 years old was 24.3%. National data have identified a small improvement in the prevalence of toothaches among Brazilians (34.8% in 2003 and 27.0% in 2010) [7], and the mean national prevalence varies from 14.5% to 34.0% [11,12,13]. It is worth noting that these data indicate a significant problem; *i.e.*, approximately one-quarter of the adult population suffers from toothache. 

Internationally, the prevalence of toothaches in adults varies from 12% to 40% [14]. However, international comparisons of studies addressing toothache prevalence must be performed with caution because of the methodological differences between studies [15].

The correspondence analysis allowed us to distinguish four groups of individuals who are suffering from a toothache. One group consisted of individuals who had less access to oral health care services (women and individuals who were multiracial or non-white, were married, had a middle school education and had the lowest incomes); the other group consisted of subjects whose toothaches were severe and caused substantial social/functional limitations (individuals aged 40 to 44 years who were married or widowed, declared themselves black or multiracial, and had a middle school education).

Both groups had low levels of schooling and were made up of either blacks or mulattoes. The difficulty regarding access may be related to the fact that individuals who are multiracial or non-white reported suffering for longer periods, and their daily activities were most affected by toothaches. There is also evidence that the use of health care services differs among social groups. While racial and ethnic minority population groups with lower education levels visit the dentist for self-perceived oral health problems, whites and individuals with higher education levels visit the dentist for preventive or follow-up visits. Thus, the presence of barriers and restricted access to dental services could result in fewer opportunities for the early detection and treatment of dental caries and other oral health problems and the prevention of pain [12]. 

Despite advances in Brazil’s public health system, toothache is the reason that 15.8% of individuals 35 to 44 year old seek dental care, according to 2010 data from Brazilian Oral Health (SB-Brazil) [7]. Although the prevalence of toothache in Brazil has decreased in recent years, it remains highly significant and may be related to the adult population’s difficulties with accessing oral health services [7]. The most recent national data showed that 7.1% of Brazilian adult population aged 35 to 44 years had never visited a dentist, 22.7% had visited a dentist more than three years ago, and 38.3% used the public dental health system [7]. 

Education and income are involved to the extent that individuals with low education and low income have a higher prevalence of oral problems [11,12]. Studies also report that the difficulties with accessing health care services, protection, and recovery noticeably grow as income decreases, which leads to greater tooth loss among patients with lower incomes [16]. The relationship between marital status and toothaches is not clear in the literature. 

Studies have shown that more women than men visit the dentist, even for routine visits, which could prevent dental complications, including toothaches [17], and that women are better able to perceive health problems and the limitations they impose [18]. However, the present study found that women had less access to oral health care services. This finding may be related to the skin colour, income, and education characteristics discussed previously. 

Regarding the severity and impact of pain on functional/social aspects, National data have shown similar results, with 15.9% of respondents reporting very weak pain, 12.4% reporting weak pain, 22.0% reporting slight strong pain, 13.2% reporting strong pain, and 36.5% reporting intolerable pain [7]. Another study showed that severe or very severe pain was present in 40.2% of respondents [19]. Obviously, different symptom characteristics (type, frequency, and severity) would have different effects on different aspects of daily performance [20]. The psychosocial impacts of oral disorders tend to vary from individual to individual, even those whose clinical symptoms are similarly severe [21]. 

The third and fourth groups indicated by the correspondence analysis were made up of individuals with higher access to oral health care services (men and individuals who were divorced, were illiterate or had a college degree, and who earned more than R$ 300.01) and those who reported low severity and a low social/functional impact (individuals who were 35 to 39 years old, white, and single and had a high school diploma), which could be explained by the fact that men have a lower biological sensitivity to stimuli and that it is more socially acceptable for men not to report pain [22]. However, the influence of gender on the sensation of toothache remains inconclusive and controversial within the general field of orofacial pain. We still do not know the extent to which differences in susceptibility and response to pain reflect characteristics of social norms for reporting pain and/or biological peculiarities related to gender, such as different biological mechanisms that drive the painful phenomenon [23]. 

As mentioned above regarding skin colour, studies have shown that whites reported less impact on their daily activities from toothache compared with individuals of other races [15]. Differences in income between blacks and whites may also be implicit because the white population in Brazil generally experience the most favourable socioeconomic conditions. 

Regarding education, it is known that higher education levels can lead to a greater appreciation of and care for the general state of health, resulting in a clearer perception of the problems and report of their impacts [16]. Other studies have found similar results [16,22]. Individuals with higher educational and income levels seek preventive health services more frequently, have better nutrition, perform more preventive self-care in general, and have less dental disease than individuals who have lower education and lower income levels [17]. Studies also indicate a link between socioeconomic status and a lower threshold for toothaches [12,23]. Although illiterate individuals were part of the group, the relatively higher incomes of these individuals would be a reason for their increased access to oral health care services and preventive and restorative treatments. 

The use of routine dental services is an important predictor of oral health, and a demand for oral health care services is the most common response to toothache [24]. However, dental treatments are still costly for the average Brazilian [25], which limits access to private services. Social and psychosocial inequalities in the use of dental services among adults, especially among low-income individuals, has been observed [26]. The proportion of dental care provided by the public health care system is 16 times higher among the poorest members of the population; however, the poorest segment of the population used the dental services three times less frequently compared with the wealthiest segment of the population [27]. 

In Brazil, the limited oral health care options for adults, a group that historically is not given priority by some models of health care, results in an accumulation of treatment needs. Inclusion of Oral Health Teams in the Family Health Strategy (Estratégia Saúde da Família-ESF) and an orientation proposed by the current National Policy on Oral Health (Smiling Brazil).aimed to change the traditional model of care in which attention is focused on priority groups (*i.e.*, school-aged children) and emergencies, instead extending the services to the entire population. 

Theoretically, this new organisation of services should encourage the regular use of dental care services by low-income adults, minimising suppressed demand and toothache [26]. However, the need for greater organisation of oral health services for elective and emergency care and expanded oral health care access for the black or mulatto adult population with a low level of schooling and low income in Brazil was demonstrated by the results of this study. 

Although the present study provides important information about toothaches in adults, the limitations, strengths, and weakness of the study should be considered. Because of the cross-sectional study design, the obtained results suggest relative hypotheses about the associated factors but have no power for causal inference. The selection of households and families as sample units was one of the study’s strengths. The survey achieved a high response rate (88.78%). Although the tool we used was not validated for researching the impact of dental pain on daily performance, the test-retest reliability of the tool was determined. In addition, Cronbach’s alpha test was also applied to all questions to verify the reliability of the components of this tool, which was generated to evaluate the impacts of toothaches.

Regarding the statistical methods used, correspondence analysis allowed us to define four profiles of individuals who were more or less affected by toothache. Because of differences in the methodology and statistical analyses employed, one limitation of the present study was the limited ability to compare these results with those of other studies that assessed the impact of toothaches on the quality of daily life or performance. Another aspect that could be considered a limitation is the high degree of subjectivity associated with investigating toothache, its perception, and its impact on daily life. However, it is clear that the contributions of the present study were greater than its limitations.

## 5. Conclusions

This study found a significant prevalence of toothaches (24.3%) in the studied population. Sixty-eight percent of the participants reported low access to dental service, 39.7% reported high pain severity, and 47.3% reported a high impact in social/functional abilities. Among the impacts on daily life, nervousness (87.2%) and eating or chewing difficulty (72.6%) were the most commonly reported. Four groups with different profiles for toothaches and associated factors were identified. Two groups exhibited greater effects of toothaches on daily life: one group consisted of individuals who reported less access to dental services (women and individuals who were multiracial or non-white, were married, had a middle school education and had the lowest incomes); the other group consisted of individuals who reported high toothache severity and a high degree of social/functional impacts (individuals who were 40 to 44 years old, married or widowed, self-declared black or multiracial, and had a middle school education). The remaining two groups were those who experienced fewer toothache-related effects on their daily life. One group consisted of individuals who reported greater access to dental services (men and individuals who were divorced, were illiterate or had a college degree, and earned more than R$ 300.01). The final group consisted of individuals who reported low toothache severity and low social/functional impacts (individuals aged 35 to 39 years who were white, single, and had a high-school education). Toothache has a negative effect on the daily lives of Brazilian adults who need treatment because of difficulties with accessing public oral health care within the country.
